# Why Do Some People Do “More” to Mitigate Climate Change than Others? Exploring Heterogeneity in Psycho-Social Associations

**DOI:** 10.1371/journal.pone.0106645

**Published:** 2014-09-05

**Authors:** José Manuel Ortega-Egea, Nieves García-de-Frutos, Raquel Antolín-López

**Affiliations:** Department of Economics and Business, University of Almería (ceiA3), Almería, Spain; The University of Adelaide, Australia

## Abstract

The urgency of climate change mitigation calls for a profound shift in personal behavior. This paper investigates psycho-social correlates of *extra* mitigation behavior in response to climate change, while also testing for potential (unobserved) heterogeneity in European citizens' decision-making. A person's extra mitigation behavior in response to climate change is conceptualized—and differentiated from common mitigation behavior—as some people's broader and greater levels of behavioral engagement (compared to others) across specific self-reported mitigation actions and behavioral domains. Regression analyses highlight the importance of environmental psychographics (i.e., attitudes, motivations, and knowledge about climate change) and socio-demographics (especially country-level variables) in understanding extra mitigation behavior. By looking at the data through the lens of segmentation, significant heterogeneity is uncovered in the associations of attitudes and knowledge about climate change—but not in motivational or socio-demographic links—with extra mitigation behavior in response to climate change, across two groups of environmentally active respondents. The study has implications for promoting more ambitious behavioral responses to climate change, both at the individual level and across countries.

## Introduction

Climate change is recognized as a major, anthropogenically-induced environmental threat, with potentially severe and far-reaching consequences for human and natural systems [1]–[2]. It is now beyond dispute that, in industrialized countries, people contribute to climate change through unsustainable high-carbon lifestyles [3]. To illustrate, in the European Union (EU), private households are directly responsible for as much as 20% of total greenhouse gas (GHG) emissions and over one-fourth of the final energy consumed [4]–[5]; substantially larger shares of EU energy use and GHG emissions are attributable in an indirect way—via consumption expenditures—to household/consumer activities [5]–[6].

Clearly, then, individuals have a central role to play in addressing climate change risks [7]. People can respond to climate change through adaptation—to potential and unavoidable climate impacts—and mitigation efforts—focused on reducing GHG emissions to prevent (or delay) further damages [3], [8]. With climate change being primarily rooted in excessive energy consumption, the public's engagement in mitigation activities is recognized as critical to achieving a sustainable future—that is, shifting towards a new, low-carbon paradigm [1], [3], [7]. Over the past two decades, increased media coverage—coupled with economic incentives, subsidies, and related interventions—has substantially raised citizens' awareness and concern about climate change, but has typically failed to induce persistent behavioral changes [1], [9]. In Europe, estimates of (some form of) personal action to mitigate climate change have ranged from 53% to 63% of the population, according to four surveys conducted between 2008 and 2011 in all EU-27 countries [10]–[11]; however, as shown elsewhere [12], there is limited engagement of most EU citizens in mitigation efforts beyond recycling [10]–[11].

Given the urgency of climate change mitigation, a profound shift is needed in personal behavior—from inaction or limited action levels—towards broader and greater levels of behavioral engagement [12]–[14]. Such *extra* behavioral responses—comprising additional mitigation actions and specific behavioral levels that go beyond what most people do—hold promise for a further incremental impact in addressing climate change. Hence, an important question arises as to what makes some people make an extra commitment (i.e., do “more”) to mitigate climate change through personal action, as compared to others.

Considerable attention has been directed at the correlates of individual pro-environmental behavior, in general, or at specific types (or subsets) of personal action—both in private and public spheres [15]–[16]. For example, past research has investigated recycling [17]–[18], reducing car use and choosing an environmentally friendly mode of transport [19]–[20], engaging in environmental citizenship [21], and many other forms of personal pro-environmental behavior. Such green behaviors have been studied through a variety of social-psychological variables and factors, including psychographics—such as environmental attitudes [15], [22], concern [23], values and motivations [24], or knowledge [25]—and socio-demographics—such as gender [26], age [27], education [28], income [29], or nationality [30]–[31].

Corresponding advances are being rapidly made in the field of climate change research, in relation to the correlates of personal mitigation (and adaptation) behavior [1]–[3], [8]. The present article aims to enhance understanding of the psycho-social correlates of extra (i.e., broader and greater) mitigation behavior in response to climate change, among the EU public. It contributes to ongoing climate change research in the following three ways. First, the authors investigate factors that relate to a person's extra behavioral engagement to address climate change, as opposed to more common (or typical) types and levels of mitigation behavior. This is important because, as advocated in previous research [14], “we must do a lot” to effectively respond to climate change. For example, if altruism is the most important motivational process associated with extra mitigation behavior, then this suggests that reinforcing altruistic values—rather than egoistic ones—is a more powerful way to support this increasingly critical action pattern. Second, the study tests for potential unobserved heterogeneity—across respondents—in the relations of psychographics and socio-demographics to extra mitigation behavior in response to climate change. For instance, by looking at the data through the lens of segmentation (i.e., through latent class regression), the analyses will clarify whether specific types of knowledge/information about climate change issues (i.e., the causes, consequences, and ways of fighting this threat) relate to similarly or differently to the breath and level of self-reported behavioral engagement (i.e., extra vs. common) in climate change mitigation. Third, through the analysis of a large-scale survey dataset of European citizens, the study increases current understanding of cross-national variations in personal mitigation behavior in response to climate change. Overall, the findings reported here should help researchers and policy-makers to promote broader and greater levels of personal response to climate change, both at the individual level and across countries.

## Extra Mitigation Behavior (in Response to Climate Change)

Pro-environmental behavior—and, more specifically, mitigation (and adaptation) responses to climate change—can be operationalized at multiple levels of analysis, such as individual, group, organizational, or regional/national levels. The focus here is on individual-level, personal mitigation behavior that, according to previous literature, can be broadly described as comprising voluntary and future-oriented behavioral responses to climate change (e.g., consumer reduction in energy consumption with mid- to long-term positive impacts on climate change) [3], [32]. Given the multi-faceted nature of personal mitigation behavior, it potentially encompasses a broad range of actions in private and public spheres of life, one-off and regular decisions, simple and more difficult steps, as well as low and high impact actions—as regards their effectiveness in mitigating climate change [1], [12], [14].

Extra mitigation behavior—the focal variable of interest in this study—is viewed as comprising additional mitigation actions (i.e., broader) and enactment levels of specific mitigation behaviors (i.e., greater) that go beyond what most people do to address climate change. A person's extra behavioral engagement to mitigate climate change is captured—and differentiated from common mitigation behavior—on a set of self-reported past/present mitigation actions spanning in-home and out-of-home settings (e.g., saving energy at home vs. reducing car use), high- and low-impact mitigation practices (e.g., energy conservation vs. recycling), one-off and frequent choices (e.g., installing solar panels in the home vs. buying seasonal, locally produced food), relating to four specific, environmentally significant domains of personal action (i.e., domestic energy/water conservation, waste reduction, eco-friendly transportation, and eco-shopping) [12]. Thus, several different types of personal action are covered so as to profile (and measure) the behavior of people who report extra behavioral engagement to mitigate climate change, as compared to others; this approach allows for sufficient variability in the difficulty, impact, frequency, and context of the specific mitigation actions being examined.

## Proposed Models of Extra Mitigation Behavior

The models tested in this study propose that environmental psychographics and socio-demographics help to explain why some people make an extra commitment to mitigate climate change through personal action, as compared to others, while testing for potential (unobserved) heterogeneity among respondent groups.

### Psychographics and extra mitigation behavior

To date, numerous studies have assessed how psychological traits relate to ecological behavior (e.g., [33]). The importance of psychographics (for pro-environmental behavior) has remained fairly stable over time [15], [34], suggesting their association with different types of climate change-motivated behavior. This study focuses first on three psychographic processes—i.e., attitudes, motivations, and knowledge about climate change (model set 1)—that likely relate to an individual's self-reported extra behavioral engagement to mitigate climate change, as compared to others.

**Attitude** is defined as the positive or negative feeling that an individual holds about a psychological object [25], [35]. The potential targets of attitudes cover a broad spectrum of discriminable aspects of the physical world, such as a physical entity, a person or group of people, an abstract concept or issue, or a behavior [36]. In the environmental literature, attitude is acknowledged as a major proximal factor for ecological intention and behavior [15], [33]. Two meta-analyses have confirmed a significant, moderate association between attitude and pro-environmental behavior, with estimated mean correlations of approximately 0.4 [15], [34]. Nonetheless, the empirical evidence has been mixed for attitudinal associations with behavior—in line with the widely reported attitude–action gap [1]. Attitude–behavior links across environmental studies are contingent on various factors, including the consideration or omission of intention as a possible mediator of this relationship [15], [35], attitude strength [37], attitude certainty and ambivalence [13], situational constraints to pro-environmental action [33], [38], and importantly, congruence in the level of specificity/generality (i.e., the scope) and time interval (e.g., simultaneous or lagged) between attitude and behavior measures [33], [39]. On the latter point, expectancy-value models recommend that attitudes be measured at the level of behavior—e.g., by measuring attitudes toward pro-environmental action, rather than attitudes toward the environment—, lagged but close in time to the measure of behavior, in order to optimize congruence in attitude–behavior measurement and to reinforce attitude associations with behavior [25], [40]–[41]. Importantly also, the analysis of attitude relations to behavior is likely to be affected by the focus on self-reports or objective outcome measures of actual behavior [41].

It is worth stressing here that investigating the attitude–action gap in climate change-motivated behavior is beyond the scope of this study, given the focus here on self-reported (instead of actual or objectively measured) mitigation behaviors, the simultaneous measurement of attitudes and behavior, and not measuring important factors such as intentions, attitudinal strength, certainty, or ambivalence. Nonetheless, attitude variables should be significantly related to EU citizens' breath and level of self-reported behavioral engagement (i.e., extra vs. common) in response to climate change, owing to: (1) the matching level of specificity/generality in attitudes (towards the threat of climate change and the role of mitigation efforts) and mitigation behavior (in response to climate change)—in a way that measurement congruence exists at the level of climate change as an environmental problem and, at least partly, at the level of behavior; (2) the close time correspondence between (present) attitudes and (past/present) self-reported behaviors; and, (3) the high degree of volitional control over performance of the behavior, which is modeled as an aggregate of specific (and highly voluntary) mitigation actions [40], [42].

Respondents with more positive/desirable attitudes, understood and measured here as positive (desirable) feelings that climate change is harmful and that mitigation actions are important, should be more likely to display extra behavioral engagement to mitigate climate change than others [12], [14]–[15]. Likewise, respondents with negative (undesirable) attitudes towards the threat of climate change (i.e., that climate change is not harmful) and the role of mitigation efforts (i.e., that mitigation behavior is not important or ineffective) should not be disposed towards extra mitigation behavior in response to climate change, as compared to others [12], [14].

**H1a.** *Positive/desirable attitudes* (towards the threat of climate change and the role of mitigation efforts) *will be positively associated with extra mitigation behavior*.

**H1b.** *Negative/undesirable attitudes* (towards the threat of climate change and the role of mitigation efforts) *will be negatively associated with extra mitigation behavior*.

**Motivation** is usually described as the driving force of behavior [25] or the “reason why a given behavior occurs” [43]. Motives can be both overt and hidden, depending on people's awareness (or not) of their motives for behavior [43]. Researchers also distinguish between primary (general) motives for a whole class of behaviors—e.g., acting in environmentally responsible ways—and selective (domain-specific) motives for particular actions, such as recycling or reducing car use [25], [43]. The present study examines the role of primary/general environmental motivations (for mitigating climate change) because of the assessment of aggregate, self-reported pro-environmental actions—i.e., people's breath and level of behavioral engagement in climate change mitigation—at a comparable level of generality [42], [44].

People may be concerned about environmental issues for several reasons [45]–[46]. Thus, previous research has explored the different types of value orientations underlying motivations for environmentally significant behavior (see [47]). Owing to the prominence of Schwartz's norm-activation model [48], most studies have differentiated between self-transcendent (altruistic) and self-enhancement (egoistic) values [47]–[49]. Stern et al. [24] further subdivided altruistic values into social-altruistic and biospheric value orientations. Similarly, Gagnon Thompson and Barton [45] drew a distinction between ecocentric and anthropocentric motives and values. Ecocentric individuals attach importance to the environment for itself and will engage in pro-environmental behavior, even if it involves some sort of sacrifice on their part [45]; this behavior pattern is largely rooted in biospheric values [24]. Anthropocentrics' actions are more deeply grounded in social-altruistic and egoistic values [24], [47]; that is, these individuals will engage in pro-environmental behavior, such as climate change mitigation behavior, only if it has positive consequences for mankind and does not diminish their quality of life or wealth [45].

Previous research suggests that, in general, pro-environmental behavior is more closely linked to biospheric values than to social-altruistic or egoistic ones [24], [29], [45]–[46]. However, not only ecocentric (biospheric) motivations, but also anthropocentric (social or egoistic) ones can relate to environmentally significant behavior [24], [29]; as shown in previous research [8], [12], non-altruistic motives—such as financial motivations—often underpin mitigation actions (e.g., energy conservation practices). There is in fact evidence of multiple motivations—i.e., altruistic and egoistic ones—for mitigation behavior [8], suggesting that both self-transcendent (altruistic) and self-enhancement (egoistic) motives may independently relate to, conflict, or converge for extra (i.e., broader and greater) behavioral engagement to mitigate climate change (among the EU public) [7], [49]–[50].

Given the mixed evidence from previous research regarding the link between self-transcendent and self-enhancement motives for and personal mitigation behavior, the issue is posed here as an explorative research question rather than as a hypothesis:

**RQ1.** *How do self-transcendent (altruistic) and self-enhancement (egoistic) motives relate to extra mitigation behavior?*

**Knowledge** of environmental issues and problems has been a significant correlate of pro-environmental awareness, moral norms, attitude, intention, and behavior [15], [25], [33]. In particular, recent meta-analytic evidence suggests the close association of environmental knowledge with pro-environmental behavior [15]. Regardless of the assumed importance of environmental knowledge (and information) as a major, but not sufficient, rational precondition for ecological action [51], its specific role in pro-environmental decision-making has long been debated [15], [34]. Information deficit approaches to behavior change—depicted largely as linear-sequential models [environmental knowledge → awareness and concern (environmental attitude) → pro-environmental behavior]—have been criticized as being too simplistic or ineffective [25]. Consistent with this, informational efforts to encourage voluntary, public engagement in climate change mitigation actions—mostly through the provision of scientifically sound information—appear to have had little success [1], [9], [13].

Multiple factors may work to strengthen knowledge/information associations with climate change mitigation action (and general pro-environmental behavior), or to cause the widely reported knowledge–action gap. First, the distinction between objective and subjective (self-reported) environmental knowledge is important; most past research implicitly assumes or explicitly states that self-assessments—used here to measure subjective knowledge about three climate change issues—serve as valid proxy measures of objective environmental knowledge (e.g., [52]–[53]), although each of these two knowledge types (objective and subjective) can be differently associated with specific pro-environmental behaviors [54]. Second, besides structural and situational constraints (see, e.g., [7]), the level and type of environmental knowledge and information have been shown to affect the strength of knowledge–behavior links [25], [40], [55]. Basic information provision is necessary for people to recognize environmental problems—e.g., to overcome the public's lack of knowledge about climate change—and consciously engage in mitigation behavior [1], [3], [25]. In contrast, an excessive amount of environmental information or very detailed technical data, concerning complex and far-reaching environmental issues such as climate change and global warming, can lead to public confusion and frustration [25], [56]. Following the distinction between declarative, procedural, and effectiveness environmental knowledge [55], knowledge about the nature and causes of environmental problems (declarative knowledge) and knowledge about ecological action strategies or “how-to” knowledge (procedural knowledge) have been especially linked to individual pro-environmental behavior [33]–[34], [55] and—importantly here—to mitigation behavior in response to climate change [8], [57]. Arguably, extra (i.e., broader and greater) personal engagement in mitigation actions should depend on the public's knowledge about the causes of climate change (and global warming)—e.g., knowledge about human contributions to climate change—and knowledge about available courses of action [8], [12]. The role of effectiveness environmental knowledge—which can be approached from two related, but distinct, angles—is somewhat more controversial. First, effectiveness environmental knowledge, when understood as knowledge about the relative ecological consequences (i.e., effectiveness) of different behavioral alternatives [55], has been shown to relate to individual pro-environmental behavior [52] and thus could be expected to be associated with extra mitigation behavior in response to climate change [8]. A second view of effectiveness environmental knowledge, referring to knowledge about the consequences of environmental problems [52]—and examined in this study in relation to self-reported climate change-motivated behavior—, has been criticized for eliciting feelings of frustration, owing to people's increased awareness of the limited impact of their actions on environmental protection [25]; thus, improving knowledge about the serious consequences of climate change—e.g., emphasizing the consequences of not engaging in mitigation behavior [52], or using fear appeals in climate change communication [9]—is not likely to result in an extra (i.e., broader and greater) level of mitigation behavior in response to climate change.

**H2.** *Both knowledge about the causes (declarative knowledge) and knowledge about the ways of fighting climate change (procedural knowledge) will be positively associated with extra mitigation behavior*.

### Socio-demographics and extra mitigation behavior

Socio-demographic variables have generally shown modest or equivocal associations with pro-environmental behavior [16], [26], [51]. In fact, some authors have argued that socio-demographics (e.g., age, sex, race, or political orientation) will be less associated with environmental concern and ecological behavior over time, due to widespread green concerns across many demographic groups [58]–[59]—particularly in Western countries [26]. This contention contrasts with recent climate change studies showing that socio-demographic variables can be significant correlates of general or specific types of personal action to mitigate climate change [3], [8], [12], [60].

Part of the between-study variation in socio-demographic associations with ecological behavior can be ascribed to methodological problems and differences across studies [61], analysis of direct vs. indirect relationships [29], [62], country-specific factors [26], or the type of behavior studied [63]. An important argument here is that some socio-demographic (background) variables may be proxies for personal capabilities—that is, the knowledge and skills necessary for particular behaviors [16], [36]. Thus, demographic variables like age, education, and income should be related to climate change mitigation efforts that depend strongly on personal capabilities [16]—i.e., mitigation actions potentially influenced by objective or subjective constraints [1], [64]. This is particularly the case of high-impact mitigation actions—i.e., energy conservation practices—which appear to be significantly associated with an individual's age (see [8]). This study evaluates the association of external socio-demographic factors with self-reported extra mitigation behavior, both at the person level—i.e., gender, age, education, and political ideology—and at the country level—i.e., country values and country wealth.

#### Gender

The evidence from prior environmental and climate change research—though far from conclusive (see [61])—suggests that women typically report greater environmental concern and involvement in environmentally significant behaviors, relative to men [3], [12], [26], [36], [65]. Specifically, women appear more likely than men to engage in private-sphere and regular pro-environmental activities in response to climate change, such as reducing waste (e.g., recycling) [12], [66] and conserving energy in the course of daily routines [12], [66].

Three theoretical explanations have been offered for gender distinctions in general environmentalism and climate change behavior. The first rationale is that traditional gender roles and socialization patterns largely underlie women's greater environmental involvement [65]. Traditional female socialization has been linked to pro-environmental behavior, owing to women's other and ecocentric value orientations [65] and caretaker role [66]. Women tend to be more attentive to the interconnections between the natural environment and things they value—e.g., other people [24]; as a result, women will be more sensitive than men to the environmental consequences of their actions [51]. The second rationale lies in the fact that, overall, women tend to judge the world as more risky [67], perceive higher levels of environmental risk [68], and thus are likely to take more pro-environmental actions than men [62]. Finally, women appear to perceive fewer (subjective and objective) constraints on personal engagement with climate change mitigation, relative to men [64].

**H3.** *Female gender will be positively associated with extra mitigation behavior.*

#### Age

There is much controversy surrounding age relations to environmental knowledge, attitudes, and behavior [26], [61]. Most studies have reported negative associations of age with environmental attitudes and concern, indicating that younger people tend to be more concerned with environmental problems such as climate change [2], [38], [61]. Less clear is the relationship between age and environmental knowledge, with age showing either non-significant or weak negative associations with knowledge about various environmental issues [26]. Also, researchers have studied the linkage between age and pro-environmental behavior with differing results—that is, age has been reported to be negatively, positively, or non-significantly related to environmentally-significant behavior (see [26]). This mixed evidence is also reflected in the study of personal mitigation behavior in response to climate change [3], [8]. In fact, asymmetric age associations with different types of action have been observed [8], [12]; to illustrate, as regards energy conservation actions, older individuals appear more likely to engage in less painful or simple energy conservation activities (e.g., buying energy-saving light bulbs or turning off unused lights), but less likely than younger people to engage in more difficult transport-related energy conservation [8], [12].

Consistent with the common negative links between age and environmental concern (i.e., attitudinal measures), Diamantopoulos et al. [26] argued that age tends to negatively correlate with *intended* ecological behavior—i.e., with intentional measures of behavior [12]; conversely, positive age–behavior linkages are typically found in studies using measures of *current* pro-environmental behavior [8], [12], [26]. Environmental attitudes and intentions may not translate into climate change mitigation behavior in younger people, partly because of their lack of necessary resources (e.g., financial means) for environmentally significant actions [26], [64]. Life-cycle and cohort effects may account as well for age differences in pro-environmental decisions [38], [61]. The life-cycle age effect points to a non-linear (inverted U-shaped) relationship between age and climate change concern; that is, highest levels of environmental concern during middle-age [38], [61]. In addition, researchers generally agree on a cohort effect, resulting from greater exposure of birth cohorts from the 1950s (or 1960s) to public discussion and concern about environmental problems—such as climate change and global warming—, compared to previous cohorts [38], [63]. Finally, “differences in time horizons in relation to climate change” [13] would be suggestive of negative age associations with personal mitigation behavior.

Taking together the available evidence—particularly the life-cycle and cohort rationales—, middle-aged European citizens are more likely to report extra mitigation behavior in response to climate change, compared to their younger and older counterparts.

**H4.** *There will be a curvilinear (inverted U-shaped) association between age and extra mitigation behavior*.

#### Education

Several studies have examined the potential role of education level as an indirect correlate—e.g., through environmental knowledge, attitudes, or concern—and direct correlate of pro-environmental behavior [26], [69]. With few exceptions (see [12], [69]), findings have been fairly consistent across studies: better-educated individuals tend to be more knowledgeable, concerned, and involved in pro-environmental activities [26], [61], [63]—including climate change mitigation actions [2]–[3], [12], [57]. Much like age and income, educational attainment may be a good proxy for personal capabilities involved in environmentally significant behavior [16], [62]. In this regard, people with more years of formal education have shown greater concern and behavioral commitment to environmental protection [26]; such individuals have access to more sources and types of information [70], and can be expected to understand highly-complex environmental issues, such as climate change, more fully than less educated citizens [26], [57], [61]. As a result, the following hypothesis is proposed:

**H5.** *Education level will be positively associated with extra mitigation behavior.*

**Political ideology** has often been employed, along with other psychological and demographic variables, to gain a deeper understanding of individual green behaviors (e.g., [8], [47], [69]). Studies including political ideology have reported very consistent results; on a left to right (liberalism–conservatism) continuum, people with left-of-center political views tend to show higher levels of concern, verbal commitment, attitudes, and environmentally significant behavior, compared to conservatives [22], [51], [63], [69]; moreover, conservative political values appear to be strongly associated with skepticism about climate change [8]. Only a few studies have not found significant associations of political orientation with pro-environmental behavior [60], [71] or climate change mitigation behavior [12]. Thus, political ideology is considered one of the most robust and stable socio-demographic correlates of environmental concern and behavior [51], [61].

**H6.** *Left-of-center political ideology will be positively associated with extra mitigation behavior*.

#### Country variations

In accordance with calls for more international research [25], cross-cultural analyses of pro-environmental and climate change-motivated behavior have garnered increased attention over the past decade [21], [31], [66], [72]–[79]. The far-reaching consequences of environmental degradation, coupled with increased (societal and governmental) environmental activism in wealthy and developing countries [80]–[81], led some authors to conclude the emergence of global environmentalism [30], [79], [82]. However, the *globalization hypothesis* has been disputed by several authors (e.g., [31], [83]); in this respect, substantial empirical evidence has accumulated in support of cross-national variations in public environmental concern and protection [46], [66], [74]–[75], [81]—including climate change mitigation efforts [78]—, both within Europe and across continents.

*Post-materialism hypothesis*. It is widely believed that international variations in pro-environmental attitudes and behavior are a consequence of different values and primary goals held across cultures [46], [75]. Particularly, Inglehart's theory of post-materialism [80] provides a prevalent value priorities approach to understanding country differences in public environmental concern (see [81]). Post-materialist theory posits that environmental concern emerges only once basic individual needs are fulfilled [80]. According to this view, people from countries with a predominant post-materialist orientation tend to be more concerned about the environment and climate change and, consequently, can be expected to make and report an extra commitment to pro-environmental and climate change mitigation behaviors [31], [80], compared to people from non-post-materialist countries. Although challenged on important points [30], [79], [82], the post-materialism hypothesis has received strong support from recent cross-cultural environmental studies [21], [31], [73], [81], [83].

**H7a.** *Post-materialist EU-27 countries will be positively associated with extra mitigation behavior*.

*Wealth hypothesis*. Cross-national variations in pro-environmental attitudes can also be explained by national differences in wealth [76], [81], [83]. Environmental concern may be an indirect consequence of wealth—i.e., mediated through post-materialist values, as asserted by Inglehart [80]—; in contrast, the prosperity/affluence hypothesis posits a direct link from wealth to environmental concern [31], [76], [83]. Regardless of small differences between the direct and indirect influence paths of wealth [31], sufficient evidence exists to suggest that citizens of wealthier nations tend to give higher priority to global environmental protection goals, compared to individuals in poorer nations [76], [80]–[81], [83]. Strong correlations have been obtained between wealth (GDP per capita) and priority/global indexes of environmental concern in the works of Franzen and colleagues—i.e., correlations of approximately 0.8, accounting for more than 50% of the cross-national variance in environmental concern (see [81]).The opposite relationship—i.e., negative correlations—has also been observed between wealth and measures of local environmental concern [30], [83]. In poorer nations, lower environmental quality and pressing ecological problems are more likely to be sources of public concern and support for local environmental protection than in rich countries [31], [81], [83]. In the present study, given the global scope of climate change, respondents from wealthier EU-27 countries are expected to report more pro-environmental attitudes and extra behavioral engagement in climate change mitigation, compared to citizens of less-wealthy nations.

**H7b.** *Wealthier EU-27 countries will be positively associated with extra mitigation behavior.*

### Unobserved heterogeneity in psycho-social associations

The implicit assumption in most environmental and climate change studies that data are collected from a single homogeneous population is, in general, unrealistic [84]. Individuals are often heterogeneous with regard to environmental psychographics—i.e., people hold different views and have different information levels of environmental problems, such as climate change—and relevant socio-demographic characteristics that can positively (or negatively) relate to pro-environmental action [85]. Such heterogeneity in public conceptualizations and preferences for mitigating climate change impacts is acknowledged as a central question in the climate change research literature [1]–[2], [9], [60]. Imposing the assumption of homogeneity—when, in fact, there is substantial (psychographic or socio-demographic) heterogeneity within the population—is likely to produce misleading inferences and biased results [84], [86]. In particular, if heterogeneity across individuals is present but ignored in regression-based studies, researchers run the risk of obtaining inconsistent model parameters and probability estimates [86].

It is important to clarify that the term heterogeneity—as used in this article—refers to both *distinct subpopulations* and *variation across individuals* [87]. In general, two forms of heterogeneity are present in data sampled from a heterogeneous population: observed and unobserved to an analyst [86]–[87]. Observed heterogeneity has been frequently dealt with—in the study of pro-environmental and climate change behavior—by the use of observed socio-economic variables (e.g., demographics like gender) that define a priori subgroups [85], [87]–[88]. However, few studies have incorporated—or attempted to uncover—unobserved heterogeneity in models of individual pro-environmental behavior or (even less so) of climate change mitigation behavior [85]–[86], [88]. With long tradition in the marketing and management literature, unobserved heterogeneity is commonly given precedence over observed heterogeneity in uncovering subpopulations (i.e., for segmentation purposes) [84], [87], probably for two main reasons. First, in uncovering unobserved heterogeneity researchers “let the data speak for itself” [87]; that is, subpopulations are unobserved by the analyst (not predefined) and have to be inferred from the data [87]. Second, unobserved heterogeneity is arguably the preferred approach for uncovering subpopulations on psychographic constructs, such as environmental attitudes and motivations [88].

Put simply, the issue of unobserved heterogeneity revolves around uncovering subgroups or segments with distinctive path model estimates [84]. In this study, potential unobserved heterogeneity is accounted for in both psychographic and socio-demographic correlates of extra personal mitigation behavior in response to climate change. By looking at the data through the lens of segmentation (i.e., response-based segmentation) [89], the findings will clarify if psycho-social associations with people's breath and level of self-reported behavioral engagement (i.e., extra vs. common) in climate change mitigation is affected by heterogeneity, or the extent to which heterogeneity exists [84].

## Methods

### Data source

The empirical analyses are performed on the cross-national dataset “Eurobarometer 69.2—Europeans' attitudes towards climate change”. A primary goal of this EU-wide survey was to investigate European citizens' climate change-related attitudes and behavior. Data were collected between March 25^th^ and May 4^th^ 2008 by TNS Opinion & Social, at the request of the European Commission, Directorate-General for Communication, Research and Political Analysis Unit. The Eurobarometer survey covers the population—aged 15 and over—of the 27 EU member states, three candidate countries (Croatia, Turkey, and the Former Yugoslav Republic of Macedonia), and the Turkish Cypriot Community. In each country, a stratified, multistage probability sampling design was used to guarantee the reliability of national and European estimates. A total of 30,170 individuals were interviewed face-to-face in their homes, and in the appropriate national language. The questionnaire addressed European citizens' self-reported attitudes, motivations, level of knowledge, and personal enactment of specific mitigation activities in response to climate change; other relevant measures were available, including materialist/post-materialist values and socio-demographic indicators, such as gender, age, education, political ideology, and country. Access to the Eurobarometer data was provided by the GESIS Data Archive for the Social Sciences (Cologne, Germany). A detailed description of the Eurobarometer dataset used here is available as electronic supplementary information (Appendix S1).

### Measurement items

Indicators of mixed scale types (i.e., categorical and continuous items) were used to measure the outcome and independent variables of the study—detailed in Tables 1 to 4.

**Table 1:** Demographic profile of respondents.

Demographic variables	Total sample (n = 30,170)	*Subsample 1*: environmentally active citizens (n = 17,233)	*Subsample 2*: environmentally inactive citizens (n = 12,937)	*χ^2^* tests: *subsample 1 vs. 2*			
				*χ^2^*	d.f.	*p*	Phi/Cramer's V
Gender				7.72	1	0.005	0.016
Male	45.6%	44.9%	46.5%				
Female	54.4%	55.1%	53.5%				
Age				150.39	5	<0.001	0.071
15–24	12.6%	10.8%	15.1%				
25–34	15.1%	15.0%	15.2%				
35–44	17.2%	18.0%	16.2%				
45–54	17.3%	17.9%	16.5%				
55–64	16.9%	17.7%	15.8%				
65+ years	20.9%	20.7%	21.2%				
Education (*age at which respondents stopped full-time education*)				528.37	5	<0.001	0.132
No full-time education	0.5%	0.2%	0.8%				
15– years	21.9%	19.7%	24.8%				
16–19	41.7%	41.3%	42.3%				
20+ years	25.3%	29.5%	19.7%				
Still studying	8.4%	7.7%	9.3%				
*Refusal/DK*	2.2%	1.6%	3.1%				
Political ideology				659.16	3	<0.001	0.148
Left/liberal	24.1%	27.1%	20.3%				
Moderate	30.2%	31.7%	28.3%				
Right/conservative	24.6%	25.3%	23.8%				
*Refusal/DK*	21.0%	16.0%	27.7%				

**Table 2:** Behavioral characterization of “extra vs. common” mitigation behavior.

Relative sizes/Mitigation actions		Segment membership probabilities a	
		Segment 1: *“common” mitigation behavior*	Segment 2: *“extra” mitigation behavior*
Relative size of segments		0.7706	0.2294
*qe6*	*Which of the following actions aimed at fighting climate change have you personally taken?*		
qe6.1	You have purchased a car that consumes less fuel, or is more environmentally friendly	0.1345	0.2973
qe6.2	You are reducing the use of your car, for example by car-sharing or using your car more efficiently	0.1651	0.4316
qe6.3	You have chosen an environmentally friendly way of transportation (by foot, bicycle, public transport)	0.2654	0.4769
qe6.4	You are reducing your consumption of energy at home (for example by turning down air conditioning or heating, not leaving appliances on stand-by buying energy efficient products such as low-energy bulbs or appliances)	0.5842	0.9254
qe6.5	You are reducing your consumption of water at home (for example not leaving water running when washing the dishes, etc.)	0.5116	0.7926
qe6.6	Where possible you avoid taking short-haul flights	0.0570	0.2958
qe6.7	You have switched to an energy supplier or tariff supplying a greater share of energy from renewable sources than your previous one	0.0537	0.1558
qe6.8	You are separating most of your waste for recycling	0.6284	0.9305
qe6.9	You are reducing the consumption of disposable items (for example plastic bags, certain kind of packaging, etc.)	0.2618	0.8318
qe6.10	You buy seasonal and local products to avoid products that come from far away, and thus contribute to CO_2_ emissions (because of the transport)	0.1500	0.6398
qe6.11	You have installed equipment in your own home that generates renewable energy (for example, a wind turbine, solar panels)	0.0411	0.1051

a Conditional (marginal) probabilities clarifying how segment-membership relates to each climate change mitigation action.

**Table 3:** Psychographic associations with extra mitigation behavior (model set 1).

Independent variables		Parameter estimates (*z*-values) a				Wald	*p*-value	Wald ( = )	*p*-value
		Class 1		Class 2					
Model 1a: **Environmental attitudes**									
qe5…	*Agreement with the following statements:*								
qe5.1	Climate change is an unstoppable process, we cannot do anything about it	−0.04	(−2.56)	−0.04	(−2.56)	6.5	0.011	*class-independent*	
qe5.2	The seriousness of climate change has been exaggerated	n.s.		−0.30	(−2.52)	6.4	0.012	-	-
qe5.3	Emission of CO2 (carbon dioxide) has only a marginal impact on climate change	−0.10	(−5.51)	n.s.		30.4	3.5e-08	-	-
qe5.4	Fighting climate change can have a positive impact on the European economy	n.s.		0.26	(1.80)	3.3	*0.072*	-	-
qe5.5	Alternative fuels, such as ‘bio fuels’, should be used to reduce GHG emissions	0.19	(2.55)	−1.07	(−3.24)	71.2	3.5e-16	20.5	6.1e-06
Model 1b: **Ecological motivations**									
qe7…	*Reasons for taking mitigation actions:*								
qe7.1	You think that if everybody changed their behavior, it will have a real impact on CC	0.14	(12.3)			152.0	6.4e-35		
qe7.2	You think that it is your duty as a citizen to protect the environment	0.08	(7.36)			54.2	1.8e-13		
qe7.3	You are very concerned about the world that you will leave for the future and young generations	0.18	(16.9)			286.6	2.8e-64		
qe7.4	You think that taking these actions will save your money	0.09	(7.78)			60.5	7.5e-15		
qe7.5	You have been directly exposed to the consequences	n.s.				0.9	*0.34*		
Model 1c: **Environmental knowledge**									
qe3…	*Well informed about:*								
qe3.1	The different causes of climate change	−0.20	(−1.36)	0.67	(2.35)	9.8	0.007	9.5	0.002
qe3.2	The different consequences of climate change	0.40	(3.06)	−0.40	(−1.33)	17.4	0.000	8.4	0.004
qe3.3	Ways in which we can fight climate change	−0.16	(−1.28)	0.41	(2.27)	7.2	0.027	7.2	0.007

a Parameter estimates represent class-specific associations, of each independent variable, with extra mitigation behavior; *z*-values in brackets.

**Table 4:** Socio-demographic associations with extra mitigation behavior (model set 2).

Independent variables	*Model 2a*				*Model 2b*				
	Parameter estimates (*z*-values) a		Wald	*p*-value	Parameter estimates (*z*-values) a			Wald	*p*-value
**Individual-level demographics**:									
Gender			57.2	3.9e-14				61.1	5.3e-15
Male	−0.083	(−7.566)			−0.086	(−7.819)			
Female	0.083	(7.566)			0.086	(7.819)			
Age			82.4	2.6e-16				87.3	2.5e-17
15–24	−0.370	(−6.924)			−0.380	(−7.132)			
25–34	−0.073	(−2.635)			−0.084	(−3.013)			
35–44	0.095	(3.848)			0.097	(3.906)			
45–54	0.163	(6.688)			0.157	(6.427)			
55–64	0.120	(4.804)			0.132	(5.276)			
65+ years	0.066	(2.595)			0.078	(3.079)			
Education			147.7	8.2e-32				157.8	5.6e-34
15– years	−0.285	(−9.638)			−0.300	(−10.097)			
16–19	−0.038	(−1.682)			−0.051	(−2.276)			
20+ years	0.125	(5.469)			0.119	(5.182)			
Still studying	0.198	(3.827)			0.232	(4.490)			
Political ideology			19.7	5.4e-05				20.3	3.9e-05
Left/liberal	0.058	(3.774)			0.064	(4.138)			
Moderate	0.008	(0.539)			−0.002	(−0.108)			
Right/conservative	−0.066	(−4.030)			−0.062	(−3.781)			
**Country-level variables**:									
Materialism/post-materialism (country groups)			542.8	1.3e-118					
Materialist countries	−0.395	(−21.925)							
Countries with mixed values	0.102	(6.610)							
Post-materialist countries	0.294	(19.231)							
Wealth (country groups)							684.6		2.1e-149
Low					−0.296	(−12.530)			
Intermediate					−0.125	(−6.311)			
High					0.421	(26.050)			

*Note*: *Models 2a and 2b* separately include each of the two country-level variables, along with the four individual-level demographics.

a Parameter estimates represent category-specific associations, of each independent variable, with extra mitigation behavior; *z*-values in brackets.

#### Measures of personal mitigation behavior

Respondents already engaged in some form of climate change-motivated activity were asked to report, on a binary nominal scale (1 =  *yes*; 0 =  *no*), whether they had undertaken each of 11 types of actions aimed at fighting climate change (see Table 2 for the list of behaviors); these activities entail different levels of mitigation difficulty, impact, and frequency in the behavioral domains of domestic energy/water conservation, waste reduction, eco-friendly transportation, and eco-shopping. An important behavioral domain not covered in this study—and reflective of high-impact mitigation behavior—is that of public sphere (public/political) environmental activism and citizenship. The focal outcome of interest, a categorical aggregate score of people's behavioral engagement (i.e., extra vs. common) in climate change mitigation, was created through segmentation analysis on all self-reported mitigation activities.

#### Measures of attitudes, motivations, and knowledge (see Table 3)

Environmental attitudes (five items) and self-reported knowledge about climate change issues (three items) were both rated on four-point scales from 1 to 4; in the attitude measures, 1 indicates *totally* disagree and 4 indicates *totally agree*; in the self-reported knowledge items, 1 denotes *not at all informed* and 4 denotes *very well informed*. Ecological motivations were measured through five possible reasons for fighting climate change; all motivation items were rated on a binary scale (1 =  *yes*; 0 =  *no*).

#### Individual-level demographics (see Tables 1 and 4)

Gender, measured as biological sex, was coded with 1 designating *male* and 2 designating *female*. Age, initially measured as a continuous variable, was divided into six age categories (coded from 1 to 6): *15*–*24, 25*–*34, 35*–*44, 45*–*54, 55*–*64*, and *65 years and over*. Education was measured by the age at which respondents stopped full-time education, and recoded into a five-category variable ranging from 0 to 4: *no full-time education*, *up to 15 years of age*, *16*–*19 years old*, *20 years and over*, and *still studying*. Political ideology was assessed through respondents' self-placement on a 10-point, left-to-right continuum; scores 1–4 were combined into 1 =  *left/liberal*; categories 5–6 into 2 =  *moderate*; and scores 7–10 into 3 =  *right/conservative*.

#### Country-level variables (see Table 4)

Participants' country is a nominal variable, with categories ranging from 1 to 33. Post-materialism, the first hypothesized explanation for country variations in citizens' self-reported behavioral engagement (i.e., extra vs. common) in climate change mitigation, was measured at the individual level through Inglehart's four-item materialist/post-materialist value battery [80]; latent class segmentation performed on these value priorities, and profiled by country, led to the classification of European countries into three materialist vs. post-materialist groups; the grouping variable was coded 1 =  *materialist countries*, 2 =  *countries with mixed values*, and 3 =  *post-materialist countries*, which closely resembles the three-class classification used in Inglehart's short post-materialism index [80], [90]. Eurostat data on GDP per capita (year 2008) was used as a proxy for EU countries' wealth; based on terciles of GDP per capita, countries were divided into three wealth groups (coded from 1 to 3): countries with *low*, *intermediate*, and *high* wealth levels.

### Statistical methodology

This paper applies latent class models (LC cluster and regression), as implemented in the Latent Gold v4.5 software, to synthesize the outcome variable and test the hypotheses linking psychographic and socio-demographic variables to the breath and level of personal mitigation behavior (i.e., extra vs. common) in response to climate change. Latent class analysis provides a powerful probabilistic approach for capturing *unobserved* heterogeneity in survey responses, and is especially useful for modeling (dependent and independent) categorical variables with varying numbers of categories [91]–[92], as in the present study. Alternative methods such as multi-group SEM allow researchers to account for *observed* heterogeneity—instead of unobserved heterogeneity—, where both the source of variation and subpopulations are known and defined a priori by the analyst. A more detailed description of statistical analysis and procedures is available as electronic supplementary information (Appendix S2).

## Results

### Descriptive profile of respondents

The sample is well-balanced in terms of gender, age, education, and political ideology (see Table 1 for results); yet, there was greater participation of female (54.4%), middle-aged (mean age  = 47.6 years), and moderately educated individuals (41.7%), with a center political orientation (30.2%). All participants were asked to report whether they had “personally taken actions aimed at helping to fight climate change”; more than half of the sample (57.1%; n = 17,233) *totally agreed* or *tended to agree* with this statement. Demographically, the subsamples of environmentally active and inactive EU citizens differed significantly (based on *χ^2^* tests), but weakly (based on association measures such as Phi and Cramer's V), in gender, age, education, and political ideology. As expected, the subsample of EU citizens already engaged in some form of climate change-motivated activity is an older, better-educated, leftist/liberal, female group, compared to environmentally inactive respondents. Subsequent analyses focused only on environmentally (i.e., climate change) active EU citizens in the year 2008 (subsample 1 in Table 1). The decision to restrict the analyses to the subsample of climate change-active citizens is consistent with this study's investigation of the correlates of “extra vs. common” personal engagement in mitigation behavior—i.e., why some people go beyond what most other environmentally active people do to mitigate climate change through extra personal action. However, it is worth raising a cautionary note about generalizing the main study's findings to environmentally inactive people; that is, what drives extra mitigation behavior may differ greatly between environmentally active vs. inactive population segments.

### Capturing extra mitigation behavior

As suggested in previous sections, LC cluster analysis was conducted on a set of 11 self-reported climate change mitigation actions (see Table 2), so as to capture and define the levels of the focal outcome variable of the study; thus, respondents were classified as showing different breath and level of personal engagement in mitigation behavior in response to climate change. The values of three segment retention criteria (BIC, AIC3, CAIC) and their percent reductions were examined for each of 10 potential cluster-solutions. These reached a minimum value for either 8 or 9 clusters; however, cluster-solutions with more than two segments were not regarded as appropriate, owing to practically insignificant percent reductions in all segment retention criteria (less than 1%), and excessive classification errors (over 10%) for the use of segment-membership as the outcome variable in subsequent regression analyses (see supplementary information—Appendix S3). The 2-cluster solution was also preferred over more complex models in terms of interpretability of the segment profiles, thus providing a higher theoretical and practical value. In sum, the results yielded an optimal solution of two segments (i.e., two differentiated behavior patterns) of EU citizens currently engaged in some form of climate change-motivated activity. This finding confirmed the distinction of two levels of “extra vs. common” personal engagement in mitigation behaviors.

Latent class sizes and levels of engagement in climate change mitigation behavior were substantially unbalanced between the two differentiated segments; *segment 1* (extra mitigation behavior) and *segment 2* (common mitigation behavior) respectively accounted for 77.1% and 22.9% of environmentally active respondents. The observations were reweighted to correct for potential biases in the latent class (segment) sizes (see Appendix S1 for details); nonetheless, the comparison of reweighted and unweighted LC cluster analyses revealed very similar results. As detailed in Table 2, respondents in segment 2 reported greater participation in mitigation efforts, compared to EU citizens showing more common types and levels of mitigation behavior (and classified into segment 1), in areas such as separating garbage for recycling (93.1% vs. 62.8%), and reducing the consumption of energy (92.5% vs. 58.4%) and water (79.3% vs. 51.2%) at home. Segment profiles differed even more in anti-shopping actions; a large majority of respondents in segment 2 claimed to be reducing their consumption of disposable items (83.2%), and avoiding products that come from far away-places (64.0%), compared to much lower shares in segment 1. The less common pro-environmental behaviors, both in the segments of people reporting extra and common mitigation behavior, refer to installing renewable energy systems in the household, switching to a greener energy supplier or tariff, and (where possible) avoiding taking short-haul flights. Overall, the profile of segment 2—and its comparison with that of segment 1—is largely in accordance with the definition of extra mitigation behavior in response to climate change given here: broader and greater engagement levels across all the specific mitigation actions and behavioral domains being examined (i.e., domestic energy/water conservation, waste reduction, eco-friendly transportation, and eco-shopping), compared to people showing more common mitigation behavior to address climate change.

### Regression results

Following LC cluster (segmentation) analysis, respondents' segment membership—i.e., a two-level categorical measure differentiating extra from common behavioral engagement in climate change mitigation—was treated as the outcome variable in two sets of LC regression models. Respectively, model sets 1 and 2 examine how environmental psychographics and socio-demographics relate to the breath and level of self-reported personal mitigation behavior in response to climate change, while exploring the extent of unobserved heterogeneity.

#### Psychographic correlates (model set 1)

The psychographic variables assessed in *model set 1* include respondents' self-reported knowledge (about the causes, ways of fighting, and consequences of climate change), positive/desirable and negative/undesirable attitudes (towards the threat of climate change and the role of mitigation efforts), and altruistic and egoistic ecological motivations (for mitigating climate change). Knowledge, attitude, and motivation associations were first examined in separate models, so as to ascertain the level of heterogeneity (if any) in the relationship of each subset of psychographic variables to the breath and level of self-reported personal mitigation behavior. Within the range of 1 to 3 latent classes, *2-class* solutions were deemed optimal in “attitude” and “knowledge” models (models 1a and 1c, respectively)—thus suggesting that unobserved heterogeneity exists in how knowledge and attitude variables relate to extra mitigation behavior, across two subgroups of environmentally active EU citizens; conversely, the *1-class* solution of homogeneity was preferable in the “motivational” model (model 1b). For a detailed description of the criteria supporting the model comparison and selection process, please see supplementary information (Appendix S3).

As an important matter of clarification at this point, the following should be noted: (1) *1-class* models entail the existence of a similar (perfectly homogeneous) pattern of association between the examined correlates with extra mitigation behavior for all environmentally active respondents; (2) *2-class* models imply that the correlates show a different pattern of association with extra mitigation behavior between two subgroups of environmentally active respondents.

All variables analyzed in the “attitude model” (model 1a) had significant links to the breath and level of self-reported personal mitigation behavior (see Table 3). For the most part, attitude associations were class-dependent—that is, four of the five attitude variables under investigation (items qe5.2 to qe5.5) behaved differently across two subgroups of environmentally active respondents. In the largest subgroup (class 1), as expected, positive/desirable attitude items (qe5.5) linked positively, and negative/undesirable attitude items (qe5.1 and qe5.3) negatively, to extra mitigation behavior. In the smallest subgroup (class 2), negative/undesirable attitude items (qe5.1 and qe5.2) were also, as hypothesized, negatively linked to extra mitigation behavior; the findings were mixed for the positive/desirable attitude items in class 2—that is, showing positive and negative associations (respectively, items qe5.4 and qe5.5) with extra mitigation behavior. In summary, the trends observed here for attitude items—despite heterogeneity across two groups environmentally active EU citizens—provide substantial support for hypotheses H1a and H1b positing that positive/desirable attitudes (towards the threat of climate change and the role of mitigation efforts) would be associated positively, and negative/undesirable attitudes negatively, with some people's extra mitigation behavior in response to climate change, as compared to others.

In the 1-*class* “motivational model” (model 1b), almost all tested variables were significantly associated with the breath and level of self-reported personal mitigation behavior—except for one motivation item: qe7.5, *you have been directly exposed to the consequences of climate change* (Wald  = 0.92; *p* = 0.34)—a finding that relates to the literature on personal experience of and behavioral responses to climate change (see the Discussion section). The other four altruistic and egoistic motivational variables (items qe7.1 to qe7.4) were significant positive correlates of extra mitigation behavior. The findings stressed the greater importance of respondents' social-altruistic motivations in understanding why some people engage in extra mitigation behavior to address climate change, as compared to others; two such social-altruistic items (qe7.3 and qe7.1) ranked first and second, respectively, in order of statistical significance (see Table 3). These results shed light on RQ1, by showing the existence of both self-transcendent (altruistic) and self-enhancement (egoistic) motives for some people's extra mitigation behavior, as compared to others, and that self-transcendent (altruistic) motives can be expected to show greater positive (desirable) associations than egoistic ones.

The three knowledge variables (about climate change issues) tested in the “knowledge model” (model 1c) were significant, class-dependent correlates of the breath and level of self-reported personal mitigation behavior (see Table 3). Interestingly, the associations of environmental knowledge items were all in the opposite direction across two classes of environmentally active respondents. In the largest subgroup (class 1), only item qe3.2, *knowledge about the consequences of climate change*, was positively associated with extra behavioral engagement to mitigate climate change; conversely, better *knowledge about the causes* (item qe3.1) and *ways of fighting climate change* (item qe3.3) was linked to extra mitigation behavior in the smallest segment (class 2). These findings are considered to partially support H2, in that both knowledge about the causes and ways of fighting climate change positively relate to extra mitigation behavior in response to climate change, but only in the comparatively small segment of more environmentally engaged EU citizens. Contrary to the authors' expectations, *knowledge about the consequences* of climate change may relate to extra mitigation efforts (in response to climate change) for the majority of environmentally active EU citizens.

Overall, the findings of the three related psychographic models (model set 1) provide evidence of unobserved heterogeneity in attitude and knowledge correlates—across two subgroups of environmentally active citizens—, and homogeneity in motivational correlates of people's breath and level of behavioral engagement (i.e., extra vs. common) in mitigation behavior to address climate change. Because of different number of *latent classes* in the “motivational model” (1-*class* model)—compared to the “attitude model” and the “knowledge model” (2-*class* models), these three types of psychographic variables were not entered into a single model simultaneously.

#### Socio-demographic correlates (model set 2)

Preliminary inspection of separate socio-demographic associations with the breath and level of personal mitigation behavior suggested the consideration of *1-class* models of homogeneity as optimal solutions across the variables under investigation. Thus—unlike in the regression model set 1—, individual and country-level socio-demographics could be entered jointly into LC regression models. Two different specifications of *model set 2* (models 2a and 2b) were then tested to examine the relationships of four individual-level demographics (gender, age, education, and political ideology) and two country-level variables (materialism/post-materialism and wealth) with EU citizens' extra (vs. common) behavioral engagement in climate change mitigation. In models 2a and 2b, each country-level variable was separately investigated, along with individual-level demographics. Within the range of 1 to 3 latent classes, *1-class* solutions were deemed optimal in the socio-demographic models 2a and 2b, thus revealing homogeneity in socio-demographic associations with extra mitigation behavior (see Appendix S3 for details).

All tested socio-demographic variables were significantly associated with the breath and level (extra vs. common) of climate change mitigation behavior in the final, 1-*class* versions of models 2a and 2b (see Table 4). Country-level variables were most significantly associated with extra mitigation behavior, with wealth ranking first and materialism/post-materialism second in statistical significance. The post-materialism hypothesis for explaining country variations in EU citizens' extra (vs. common) mitigation behavior was tested and strongly supported in model 2a; accordingly, the post-materialist country group showed the strongest positive association with extra personal engagement in mitigation behavior. Further, the results of model 2b confirmed the importance of the wealth hypothesis; only the wealthiest country group showed a positive association with extra behavioral engagement to mitigate climate change. In summary, the previous findings yielded full support for the two hypotheses involving country-level variables (support for H7a and H7b).

The parameter estimates for individual-level demographics were almost identical in the alternative model specifications 2a and 2b (all significant at *p*<0.001). As expected, female gender was positively, although somewhat weakly, related to extra mitigation behavior to address climate change (support for H3). As suggested by the visual inspection of findings in Table 4, a curvilinear-like or “plateau” relationship appeared to exist between age and the breath and level of personal mitigation behavior, with the 45–56 age group showing the highest association with extra mitigation action. A complementary test of non-linearity was performed to clarify the precise form of age associations; for this purpose, the age variable—with linear ranges and of equal span—was converted into a quasi-interval scale and the authors tested whether a linear, curvilinear, or quadrilateral relationship was the best way to describe age relations to extra mitigation behavior. As expected, and detailed in the supplementary information (Appendix S4), a curvilinear function provided the optimal fit to the age data. Thus, there was considerable support for hypothesis H4 of a curvilinear (inverted U-shaped) relationship between age and EU citizens' extra behavioral engagement in mitigation behavior—mostly grounded in the interplay of cohort and life-cycle age effects on pro-environmental (climate change) concern and behavior. Regarding education, a positive association was identified between the age at which respondents stopped full-time education and extra mitigation behavior—which is fully supportive of H5. Finally, the results supported the hypothesis (H6) that people with a leftist/liberal political orientation would display extra (i.e., broader and greater) behavioral engagement in climate change mitigation, compared to right-wing/conservative environmentally active respondents.

## Discussion

A fundamental shift is needed towards broader and greater levels of behavioral engagement in the population that provide further incremental benefits in addressing climate change. The cross-national research reported here aimed to enhance understanding of what makes some people make an extra commitment (i.e., do “more”) to mitigate climate change through personal action, as compared to others. The authors assessed the role of psychographics, individual and country-level socio-demographics, and the extent of (unobserved) heterogeneity in an individual's extra behavioral engagement to address climate change.

In line with past environmental research (e.g., [93]–[94]), segmentation analysis revealed two heterogeneous (and differentiated) action patterns in response to climate change, among environmentally active EU citizens. These findings validated the a priori, intuitive two-level distinction of “extra vs. common” mitigation behavior in the focal variable of interest. As expected, engaging extra to mitigate climate change through personal behavior, compared to more common (or typical) mitigation behavior, was reflected in broader and greater self-reported engagement in all specific mitigation endeavors—entailing different levels of difficulty, impact, and frequency—and behavioral domains being examined [8], [14].

The definition and measurement in aggregate of extra mitigation behavior allowed the analysis of factors that could influence broader and greater levels of mitigation behavior in a variety of settings. Overall, the findings reinforce earlier evidence that environmental psychographics (i.e., attitudes, motivations, and knowledge about climate change) are more associated with personal mitigation efforts in response to climate change than socio-demographics [12].

A central question in this study was: Is there heterogeneity in psycho-social correlates of extra mitigation behavior? The findings revealed that unobserved heterogeneity significantly affects how attitude and knowledge variables relate to extra (vs. common) personal behavior to mitigate climate change, but does not affect the links with motivations or socio-demographics. These results warn of the risk of ignoring the potential presence of unobserved heterogeneity in the analysis of attitudinal and knowledge associations with climate change-motivated behavior—i.e., researchers run the risk of obtaining biased or inconsistent results.

Positive/desirable and negative/undesirable attitude variables were significantly associated with extra mitigation behavior. Unlike most expectancy-value models [15], [34], [40], attitudes were not measured here only at the level of behavior; however, the congruent level of specificity/generality in attitudes (towards the impact of climate change and mitigation efforts) and self-reported mitigation behavior (in response to climate change), the close time correspondence between attitudes and behaviors, coupled with the assessment of aggregate mitigation behavior—i.e., offsetting differences in behavioral control across 11 types of mitigation actions—, is likely to have reinforced the significance attitude relations to behavior [33], [39]–[40]. Overall, the findings emphasize the importance of building citizens' positive/desirable attitudes toward climate change issues (i.e., feelings that climate change is harmful and that mitigation actions are important), and reducing negative/undesirable ones (i.e., feelings that climate change is not harmful and that mitigation behavior is not important or ineffective)—but differently for two subgroups of environmentally active EU citizens—, so as to effectively promote broader and greater levels of engagement in mitigation action in response to climate change.

An important finding concerned the role of self-reported knowledge about climate change issues. Respondents' level of knowledge about the causes and ways of fighting climate change—on the one side—, and knowledge about the consequences of climate change—on the other side—were all significantly, but inversely, related to extra mitigation behavior, across two (environmentally active) respondent subgroups. In contrast with most previous studies [8], [33], [55], informing the public about the consequences of climate change appears to be more useful, than informing about the causes and ways of fighting climate change, in promoting extra mitigation behavior for the majority of environmentally active citizens—i.e., people that currently display limited action levels in response to climate change. Thus, not all types of climate change messages and information can be assumed to be equally effective in promoting extra mitigation efforts in the population.

In line with previous studies (e.g., [50]), both altruistic and egoistic motivations were positively associated with extra (vs. common) personal behavior to mitigate climate change for all environmentally active respondents. Thus, interventions aimed at encouraging citizens to undertake more ambitious mitigation efforts should appeal to both altruistic and egoistic ecological motives and values. Nonetheless, as suggested in earlier work (e.g., [95]), the results warn that motivational influences on pro-environmental behavior tend to vary in strength (and importance). The only motivational variable not significantly associated with the extra mitigation behavior concerned respondents' direct exposure to the consequences of climate change. Such a finding (as noted earlier) adds to the inconclusive evidence in the literature on the association of first-hand experience of climate change consequences and personal engagement in mitigation behavior [96]. There is evidence to suggest that personal experience of different types of climate change consequences (e.g., flooding vs. air pollution) are likely to elicit different personal behavioral responses [97]—an issue that could not be addressed here and requires further investigation.

This study further suggests that—despite equivocal evidence from previous environmental studies (e.g., [16], [26], [51])—, a variety of individual and country-level socio-demographics are significant correlates of extra mitigation behavior in response to climate change. Country-level variables were most significantly related to respondents' level of climate change mitigation behavior. In line with Franzen's work (e.g., [31], [81]), strong support was obtained for the post-materialism and wealth hypotheses—i.e., broader and greater levels of behavioral engagement to mitigate climate change in EU countries with predominant post-materialist values and in wealthier nations. These findings exemplify the cultural and economic underpinnings of personal mitigation behavior in response to climate change. Accordingly, climate change campaigns encouraging people to do “more” or “a lot” through environmentally responsible behaviors should be tailored to each cultural and wealth EU country group.

The results for individual-level demographics also support the contention that demographic variables continue to be significant correlates of pro-environmental behavior [22], [57], [60], [63]. As hypothesized, the findings confirm the need to encourage broader and greater mitigation responses to climate change, especially among men, conservatives, less educated individuals, and both the youngest and oldest population groups. The curvilinear (inverted U-shaped) link between age and extra mitigation behavior warn environmental researchers of potential non-linear associations of socio-demographics with pro-environmental (climate change) behavior. Thus, researchers are advised to use statistical methods which can detect the presence of potential non-linearities (and heterogeneity), such as latent class analysis or neural networks.

## Limitations and Recommendations for Further Study

The present study has a number of limitations that should be addressed in future work. First, the use of secondary survey data—i.e., the Eurobarometer dataset—limited the scope of the research questions and the operationalization of the study variables. Despite the advantages of Eurobarometer data, such as providing a rich, cross-national source of information—with minimum time and financial investment—, its use inhibited the analysis of other relevant correlates of extra mitigation behavior, such as behavioral intentions, subjective norm, or perceived behavioral control [40]. Thus, future studies should consider including unmodeled factors such as citizens' perceptions of self-relevance or involvement with climate change and global warming issues, using alternative and refined measures of psychographics (e.g., attitudes toward the behavior) and behavior (e.g., lagged or future mitigation action), and assessing situational deterrents (and enhancers) of broader and greater levels of behavioral engagement to mitigate climate change. In addition, the important behavioral domain of public sphere (public/political) environmental activism and citizenship, and reflective of high-impact mitigation behavior—that could not be covered in this study—should be accounted for in the future.

Second, this study is based on self-reported (instead of actual or objectively measured) mitigation behavior and the correlates of interest. On the one hand, the focus on self-reported mitigation behavior, coupled to the simultaneous measurement of attitudes and behavior, did not allow the analysis of attitude–action gap in climate change-motivated behavior—an important issue that warrants further investigation through comparison of self-reports and objective measures of actual behavior. On the other hand, it is possible that social desirability biases may have played a role, leading some respondents to overstate their knowledge about climate change issues and their engagement in mitigation actions. Past research has shown the correlation and redundancy between measures of perceived and objective environmental knowledge, but also their potentially differential associations with pro-environmental behaviors [54]. The disparity between perceived and objective knowledge (and their relationships) deserves future attention in studies on climate change-motivated behavior. As regards mitigation behavior, social desirability arguably represents a minor problem in light of the analysis of a representative, non-student sample of environmentally active EU citizens, and the satisfactory correspondence of the self-report measures used in this study to *past/present* behavior—measured as *yes/no* present enactment of specific mitigation actions. Non-student samples, people with high scores of ecological behavior, and measures of past/present (rather than *intended/future*) pro-environmental behavior are likely to be less affected by social desirability biases [52].

Third, the analyses reported here were restricted only to participants already engaged in some form of climate change-motivated activity (i.e., environmentally active citizens). This decision was consistent with this study's investigation of the correlates of “extra vs. common” to mitigate climate change through personal behavior. In pursuing this objective, respondents not reporting any action on climate change did not provide relevant information on the focal variable (extra mitigation behavior) and its underlying motivations. Yet, the exclusion from the analyses of environmentally inactive respondents should be acknowledged as a significant methodological and practical limitation that cautions against generalizing the present study's findings to environmentally inactive people—around 40% of EU-27 citizens [10]–[11]; this is important because environmentally active vs. inactive population segments are likely to differ in what drives extra (i.e., broader and greater levels of) mitigation behavior. The shortcomings of the current approach could be overcome in future studies that extend understanding of what makes people do “a lot” to mitigate climate change beyond the more receptive (and arguably less challenging) population segment of environmentally active citizens. Such broader investigations would make additional progress toward ambitious goals in the public's behavioral engagement to mitigate climate change.

Fourth, this study examined only direct (psychographic and socio-demographic) correlates of extra climate change mitigation behavior. However, full understanding of the complex and dynamic mechanisms involved in such environmentally significant behavior requires that direct, indirect, and moderating influences be considered. Statistical methodologies such as structural equation modeling (SEM) or partial least squares (PLS) are most appropriate to unravel the interplay between internal and external correlates of broader and greater levels of personal response to climate change [69]—for instance, by placing the variables under the nomological structure of expectancy-value models [40]. If possible, future research should explore lagged effects of knowledge, motivations, and attitudes on intentions and future behavior.

Future research should continue to address the important topic of unobserved heterogeneity in pro-environmental decisions [93]–[94]. Particularly, additional empirical evidence is needed to verify the heterogeneous (class-dependent), psychographic links to personal mitigation behavior in response to climate change, observed in this study. The significance and strength of country-level associations warrant additional international analyses of pro-environmental behavior in response to climate change. Also, the interaction between individual-level psychographics (e.g., knowledge and attitudes) and countries' wealth and post-materialism levels deserves closer attention in relation to personal mitigation behavior. Preliminary analyses (not reported here but available upon request) show that, amongst post-materialist and wealthier EU countries, the findings tend to be more consistent with previous literature [8], [33], [55]—e.g., there is greater presence of the segment of environmentally active people for whom knowledge about the causes and ways of fighting climate change relates to extra behavioral engagement to mitigate climate change, and positive/desirable and negative/undesirable attitudes are significantly associated (positively and negative, respectively) with extra mitigation behavior. Certainly, wealth and post-materialism are not the only valid approaches to explaining country differences in climate change mitigation behavior. Other potentially relevant cultural dimensions for cross-national environmental studies include harmony [21]; individualism, long-term orientation, and locus of control [77]; and traditional and altruistic values [72]. Country and regional heterogeneity in environmental policy and legislations are also likely to account for national differences in public mitigation behavior. Multilevel analysis seems to be most appropriate for environmental research questions involving variables from different levels—e.g., individual and country-level influences on climate change-motivated behavior.

## Concluding Remarks

As a result of the variety of variables analyzed in relation to extra (vs. common) personal behavior to mitigate climate change, the following can be concluded:

1. A profound shift is needed in personal behavior—from inaction or limited action levels—toward extra (i.e., broader and greater levels of) behavioral engagement to mitigate climate change.
2. The population segments of environmentally active and inactive EU citizens differed significantly, but weakly, in demographic terms.
3. Environmentally active citizens—the population segment under study—can be differentiated in two intuitive categories: people reporting “extra” vs. “common” behavioral engagement to mitigate climate change.
4. Both psychographics and (individual and country-level) socio-demographics help to explain why some people make an extra commitment to mitigate climate change through personal action, as compared to others.
5. Psychographics tend to show greater association than socio-demographics with extra mitigation behavior in response to climate change.
6. There is heterogeneity in the associations involving attitude and knowledge variables, whereas homogeneity exists in the links of motivations and socio-demographics with extra mitigation behavior.
7. The findings draw attention to the importance of potential non-linearities in socio-demographic correlates.
8. The study has implications for promoting more ambitious behavioral responses to climate change, both at the individual level and across countries.

## Supporting Information

Appendix S1 Detailed description of Eurobarometer 69.2.
(DOCX)

Appendix S2 Supplementary description of statistical analysis.
(DOCX)

Appendix S3 Criteria supporting the model comparison and selection process: LC cluster and regression models.
(DOC)

Appendix S4 Complementary test of non-linear age associations.
(XLS)
