# Supplementary material for: Why Do Some People Do “More” to Mitigate Climate Change than Others? Exploring Heterogeneity in Psycho-Social Associations
Source: PLoS One. 2014 Sep 5;9(9):e106645. doi: 10.1371/journal.pone.0106645 (PMC4156351; doi:10.1371/journal.pone.0106645)
Supplement: Appendix S1 — Detailed description of Eurobarometer 69.2. (DOCX) [file pone.0106645.s001.docx]

**Appendix S1. Detailed description of Eurobarometer 69.2**

The total sample of 30,170 respondents was stratified by country, with very homogeneous national sample sizes (with few exceptions, approximately 1000 per country) that are disproportional to the actual population sizes (see Table S1). This sampling strategy is standard in international studies—based on individual level-data—seeking to optimize the accuracy of both national and cross-national results. In the Eurobarometer 69.2, sufficiently large national subsamples allow single-country analyses and cross-national comparisons with adequate precision. However, similar country sample sizes could seriously bias the results and conclusions on the entire European dataset. Thus, reweighting is recommended to ensure that the pooled sample is representative of the reference population. The Eurobarometer database provides a weighting variable (population size weight) that corrects for discrepancies between national samples and population sizes, while also guaranteeing that country samples are representative in socio-demographic terms. Table S1 shows the average values of the weighting variable per EU country, the national sample and population distributions (absolute sizes and proportions), and the overall sampling error of the survey.

**Table S1. Descriptive statistics for country samples and populations**

| **Country** | **Population size and proportion** *(citizens aged 15+)* ^a^ | | **Sample size and proportion** ^b^ | | **Average population weight** |
| --- | --- | --- | --- | --- | --- |
| Belgium | 8,786,805 | (1.94%) | 1003 | (3.32%) | 0.5823 |
| Bulgaria | 6 ,647,375 | (1.46%) | 1000 | (3.31%) | 0.4419 |
| Czech Republic | 8,571,710 | (1.89%) | 1014 | (3.36%) | 0.5619 |
| Denmark | 4,432,931 | (0.98%) | 1005 | (3.33%) | 0.2932 |
| Germany West | 51,872,952 | (11.4%) | 1027 | (3.40%) | 3.3575 |
| Germany East | 12,673,144 | (2.79%) | 507 | (1.68%) | 1.6616 |
| Estonia | 887,094 | (0.20%) | 1006 | (3.33%) | 0.0586 |
| Greece | 8,691,304 | (1.91%) | 1000 | (3.31%) | 0.5777 |
| Spain | 38,536,844 | (8.49%) | 1033 | (3.42%) | 2.4798 |
| France | 46,425,653 | (10.2%) | 1040 | (3.45%) | 2.9674 |
| Ireland | 3,375,399 | (0.74%) | 1004 | (3.33%) | 0.2235 |
| Italy | 48,892,559 | (10.8%) | 1022 | (3.39%) | 3.1801 |
| Cyprus | 638,900 | (0.14%) | 504 | (1.67%) | 0.0843 |
| Turkish Cypriot Comm. | 143,226 | (0.03%) | 500 | (1.66%) | 0.0190 |
| Latvia | 1,444,884 | (0.32%) | 1008 | (3.34%) | 0.0953 |
| Lithuania | 2,846,756 | (0.63%) | 1021 | (3.38%) | 0.1853 |
| Luxembourg | 388,914 | (0.09%) | 501 | (1.66%) | 0.0516 |
| Hungary | 8,320,614 | (1.83%) | 1000 | (3.31%) | 0.5531 |
| Malta | 335,476 | (0.07%) | 500 | (1.66%) | 0.0446 |
| Netherlands | 13,017,690 | (2.87%) | 1041 | (3.45%) | 0.8312 |
| Austria | 7,004,205 | (1.54%) | 1000 | (3.31%) | 0.4656 |
| Poland | 32,155,805 | (7.08%) | 1000 | (3.31%) | 2.1375 |
| Portugal | 8,080,915 | (1.78%) | 1001 | (3.32%) | 0.5366 |
| Romania | 18,246,731 | (4.02%) | 1019 | (3.38%) | 1.1903 |
| Slovenia | 1,729,298 | (0.38%) | 1003 | (3.32%) | 0.1146 |
| Slovakia | 4,316,438 | (0.95%) | 1085 | (3.60%) | 0.2645 |
| Finland | 4,353,495 | (0.96%) | 1004 | (3.33%) | 0.2882 |
| Sweden | 7,562,263 | (1.67%) | 1007 | (3.34%) | 0.4992 |
| United Kingdom | 50,519,877 | (11.1%) | 1306 | (4.33%) | 2.5714 |
| Croatia | 3,734,300 | (0.82%) | 1000 | (3.31%) | 0.2482 |
| Turkey | 47,583,830 | (10.5%) | 1003 | (3.32%) | 3.1536 |
| F.Y.R. of Macedonia | 1,648,012 | (0.36%) | 1006 | (3.33%) | 0.1089 |
| *Totals* | 453,865,399 | (100%) | 30,170 | (100%) | - |
| ^a^ *Source*: Eurostat (2008).  ^b^ Overall sampling error = 0.6%. | | | | | |
