# Supplementary material for: Why Do Some People Do “More” to Mitigate Climate Change than Others? Exploring Heterogeneity in Psycho-Social Associations
Source: PLoS One. 2014 Sep 5;9(9):e106645. doi: 10.1371/journal.pone.0106645 (PMC4156351; doi:10.1371/journal.pone.0106645)
Supplement: Appendix S2 — Supplementary description of statistical analysis. (DOCX) [file pone.0106645.s002.docx]

**Appendix S2. Supplementary description of statistical analysis**

**LC cluster (segmentation) method**. LC cluster analysis is used here to capture extra mitigation behavior to address climate change—and to differentiate it from more common or typical mitigation behavior—, among EU citizens currently engaged in some form of climate change-motivated activity. A key, but often arbitrary, decision in cluster analysis concerns the determination of the number of patterns (clusters, segments, classes, or groups); unlike other methods, the LC approach provides formal criteria for establishing the optimal number of segments. Segment retention criteria, such as BIC, AIC3, and CAIC, will be used to assess the relative fit and parsimony of alternative LC models. Applying the *minimum-value* rule, cluster models with lower values in the previous information criteria should be preferred. However, large sample sizes increase the tendency of information criteria towards overfitting, thus indicating an excessive number of segments as the appropriate solution. In that case, significant percent reductions in segment retention indices will be required between competing cluster models. Low classification error is also a necessary condition for optimal model selection, especially given the purpose of the segmentation analysis in this study—i.e., aggregating the focal outcome variable; the classification error statistic estimates the proportion of misclassified cases by “modal assignment”—i.e., the class having the highest posterior membership probability.

**LC regression method**. Respondents’ segment membership (i.e., “extra vs. common” mitigation behavior segments) is treated as the outcome variable in LC regression models. Unlike standard regression, latent class regression will account for potential unobserved heterogeneity and non-linearities in estimating associations of psychographics and socio-demographics with extra mitigation behavior. Because of the possibility of uncovering heterogeneity, the segment retention criteria previously described (BIC, AIC3, CAIC) will also be useful in LC regression to establish the optimal number of underlying classes/segments. In particular, in regression-based segmentation and more complex models, AIC3 has been shown to outperform other segment retention criteria. Finally, the pseudo *R*^2^ statistic is used to assess the prediction performance of alternative LC regression models; that is, the pseudo *R*^2^ can be interpreted as the proportion of variation (in the outcome) explained by the correlate variables in the LC model, with values ranging from 0 to 1.

**Reweighting method**. Two-step reweighting is applied in all LC analyses to correct for the differential national populations of European citizens; in the first step, unweighted analysis yields more stable (more efficient) parameter estimates defining the latent classes; in step two, weighted analysis corrects for potential biases in the latent class sizes and, if included, covariate effects using sampling weights—i.e., the population size weights available in the Eurobarometer database.
