# Supplementary material for: Why Do Some People Do “More” to Mitigate Climate Change than Others? Exploring Heterogeneity in Psycho-Social Associations
Source: PLoS One. 2014 Sep 5;9(9):e106645. doi: 10.1371/journal.pone.0106645 (PMC4156351; doi:10.1371/journal.pone.0106645)
Supplement: Appendix S3 — Criteria supporting the model comparison and selection process: LC cluster and regression models. (DOC) [file pone.0106645.s003.doc]

**Appendix S3. Criteria supporting the model comparison and selection process: LC cluster and regression models**

**Table S3.1. Segment retention criteria for different LC cluster-solutions: extra mitigation behavior**

| Number of segments | BIC (LL) | Change in BIC ^a^ | AIC3 (LL) | Change in AIC3 ^a^ | CAIC (LL) | Change in CAIC ^a^ | Classification error |
| --- | --- | --- | --- | --- | --- | --- | --- |
| 1 cluster | 193901.7 | - | 193827.4 | - | 193912.7 | - | 0.0000 |
| **2 clusters** | 187295.4 | **-3.41%** | 187139.9 | **-3.45%** | 187318.4 | **-3.40%** | **0.0896** |
| 3 clusters | 186312.7 | -0.52% | 186076.1 | -0.57% | 186347.7 | -0.52% | 0.1818 |
| 4 clusters | 186143.6 | -0.09% | 185825.8 | -0.13% | 186190.6 | -0.08% | 0.2793 |
| 5 clusters | 186085.5 | -0.03% | 185686.7 | -0.07% | 186144.5 | -0.02% | 0.3176 |
| 6 clusters | 185889.1 | -0.11% | 185409.2 | -0.15% | 185960.1 | -0.10% | 0.3985 |
| 7 clusters | 185834.4 | -0.03% | 185273.3 | -0.07% | 185917.4 | -0.02% | 0.3987 |
| 8 clusters | *185806.7* | -0.01% | 185164.5 | -0.06% | *185901.7* | -0.01% | 0.4041 |
| 9 clusters | 185814.1 | 0.004% | *185090.7* | -0.04% | 185921.1 | 0.01% | 0.3941 |
| 10 clusters | 185914.1 | 0.05% | 185109.6 | 0.01% | 186033.1 | 0.06% | 0.4080 |
| ^a^ Changes in BIC, AIC3, and CAIC refer to the previous number of clusters; the lowest values are printed in italics and underlined.  ^b^ The values supporting the appropriateness of the *2-segment* solution are printed in boldface. | | | | | | | |

**Table S3.2. Selection criteria for competing ‘psychographic’ (LC regression) models: models 1a (attitudes), 1b (motivations), and 1c (knowledge)**

| Tested models | Log-likelihood (LL) | BIC (LL) | AIC3 (LL) | CAIC (LL) | Pseudo *R*^2^ |
| --- | --- | --- | --- | --- | --- |
| model1a–attitudes, 1 class | -6764.6 | 13586.2 | 13547.3 | 13592.2 | 0.0240 |
| model1a–attitudes, 2 classes: 1 | -6695.5 | 13514.5 | 13430.1 | 13527.5 | 0.2319 |
| **model1a–attitudes, 2 classes: 2** | **-6695.2** | ***13475.8*** | ***13417.3*** | ***13484.8*** | **0.2322** |
| model1a–attitudes, 3+ classes | *Convergence problems and instability of parameter estimates* | | | | |
| **model1b–motivations, 1 class** | -8288.4 | ***16635.2*** | 16594.7 | ***16641.2*** | 0.0424 |
| model1b–motivations, 2 classes | -8268.2 | 16663.2 | *16575.5* | 16676.2 | 0.0928 |
| model1b–motivations, 3+ classes | *Convergence problems and instability of parameter estimates* | | | | |
| model1c–knowledge, 1 class | -8360.3 | *16759.6* | 16732.7 | *16763.6* | 0.0136 |
| **model1c–knowledge, 2 classes: 1** | **-8343.1** | 16773.9 | ***16713.3*** | 16782.9 | **0.4669** |
| model1c–knowledge, 3+ classes | *Convergence problems and instability of parameter estimates* | | | | |
| ^a^ The lowest BIC, AIC3, and CAIC values are printed in italics and underlined.  ^b^ The (*1-class* or *2-class*) models selected and the values supporting their appropriateness are printed in boldface. | | | | | |

**Table S3.3. Selection criteria for competing socio-demographic (LC regression) models: models 2a (materialism/post-materialism) and 2b (wealth)**

| Tested models | Log-likelihood (LL) | BIC (LL) | AIC3 (LL) | CAIC (LL) | Pseudo*R*^2^ |
| --- | --- | --- | --- | --- | --- |
| **m2a-dem-mat/post, 1 class** | -6854.4 | ***13842.9*** | 13750.8 | ***13856.9*** | 0.0958 |
| m2a-dem-mat/post, 2 classes | -6808.8 | 13895.4 | *13704.7* | 13924.4 | 0.2842 |
| m2a-dem-mat/post, 3+ classes | *Convergence problems and instability of parameter estimates* | | | | |
| **m2b-dem-wealth, 1 class** | -6753.9 | ***13641.8*** | 13549.7 | ***13655.8*** | 0.1100 |
| m2b-dem-wealth, 2 classes | -6711.0 | 13699.8 | *13509.1* | 13728.8 | 0.3791 |
| m2b-dem-wealth, 3+ classes | *Convergence problems and instability of parameter estimates* | | | | |
| ^a^ The lowest BIC, AIC3, and CAIC values are printed in italics and underlined.  ^b^ The (*1-class*) models selected and the values supporting their appropriateness are printed in boldface. | | | | | |
